# A systematic review and meta-analysis of phospholipids in human milk

**DOI:** 10.3389/fnut.2026.1929517

**Published:** 2026-09-23

**Authors:** Huanzhe Dong, Menglu Li, Yuling Xue, Shijie Wang, Nan Wang, Benheng Guo, Yiping Xun

**Affiliations:** 1 Nutrition Research Institute, Junlebao Dairy Group Co., Ltd., Shijiazhuang, Hebei, China; 2 College of Food and Biology, Hebei University of Science and Technology, Shijiazhuang, Hebei, China

**Keywords:** composition, human milk, lactation stage, phospholipid, systematic review

## Abstract

**Background:**

Phospholipids are important bioactive components in human milk. They are rich in unsaturated fatty acids and play crucial roles in cell signal transduction and infant intellectual development.

**Methods:**

This study quantitatively pooled concentrations of total phospholipids and their subclasses in human milk, and narratively summarized phospholipid subclass proportions across different countries. Relevant studies published up to October 2025 were searched from six databases. After systematic search, a total of 25 studies were finally included, among which 18 studies on phospholipid concentrations were subjected to meta-analysis.

**Results:**

Results from the random-effects model showed that the total phospholipid concentration was 251.60 mg/L (175.62, 327.58) in colostrum, 234.71 mg/L (171.07, 298.35) in transitional milk, 197.00 mg/L (166.08, 227.93) in mature milk. Throughout lactation, PC, SM, and PE are the major phospholipid classes in human milk. Subgroup meta-analyses indicated that detection methods were a significant effect for total phospholipids, PE, and PI concentrations within mature milk.

**Conclusion:**

This systematic review provides updated estimates of phospholipids and their subclasses concentrations in term human milk, and provides a theoretical basis for the application of phospholipids in infant formula.

**Systematic review registration:**

https://www.crd.york.ac.uk/prospero/, Identiffer CRD420261445346.

## 1 Introduction

Human milk is the optimal nutritional choice for infants aged 0–6 months (1). Among the nutritional components of human milk, fat provides 45–50% of the energy for infants with an average concentration of 3.5–4.5 g/mL, which is crucial for infant growth and development. Phospholipids account for approximately 0.2–2% of total fat in human milk (2). Although phospholipids only make up a small proportion of total lipids in human milk, when compared with triglycerides, which serve as the major energy source for organisms, phospholipids display greater biological activity and play an indispensable role in maintaining normal physiological functions. As essential structural components of all biological cell membranes, phospholipids participate in lipid digestion, absorption, and transport. Moreover, as fundamental biomolecules supporting life activities, they play critical roles in cellular metabolism and signal transduction (3–5). Research has shown that phospholipids exert beneficial effects on the infant health, including promoting brain and cognitive development, regulating infant intestinal and immune responses, and inhibiting cholesterol absorption (6–8).

Phospholipids have a hydrophobic tail and a hydrophilic head, making them an important class of amphiphilic molecules. They are composed of glycerol or sphingosine, fatty acids, phosphate, and nitrogen-containing compounds. According to the different structures of their main carbon chains, they can be mainly categorized into glycerophospholipids (GPLs) and sphingomyelin (SM). GPLs have a triacylglycerol backbone, in which the hydroxyl groups at the sn-1 and sn-2 positions of glycerol are esterified with various fatty acids, while the phosphate head group is attached to the sn-3 position of glycerol. According to the different base groups attached to the phosphate at the sn-3 position, it can be divided into phosphatidylcholine (PC), phosphatidylethanolamine (PE), phosphatidylinositol (PI), and phosphatidylserine (PS), etc. (9–11). SM has a sphingosine backbone and is directly linked to a phosphate group. In the human milk, 60–65% of the phospholipids are located in the milk fat globule membrane (MFGM), where they coexist with cholesterol, enzymes, glycolipids, and glycoproteins. PC and SM are mainly located in the outer layer of the membrane, while PE, PI, and PS are concentrated on the inner surface (12, 13). This distribution has a significant impact on microstructure of biological membranes, stabilizing the lipid core of the milk fat globules and protecting it from degradation by lipases (14).

Phospholipid subclasses differ in structure, properties and functions. Variations in the composition of fatty acyl chains and the linked phosphate groups in phospholipids exert significant effects on their physiological functions. Understanding the changes in phospholipid composition and phospholipid species during lactation is a key step in determining infant nutritional requirements. Therefore, this study summarizes the concentrations of total phospholipids and five major phospholipid subclasses in human milk. Subgroup analysis were applied to assess the differences in the phospholipid composition of human milk across various lactation stages, national economic level, geographical regions and detection methods. This meta-analysis aims to construct global reference intervals for human milk phospholipids and characterize their variations with lactation stage and geography.

## 2 Materials and methods

### 2.1 Literature search and selection

This study searched English and Chinese databases, including China National Knowledge Infrastructure (CNKI), Wanfang Database, VIP Database, PubMed, Web of Science and Sciencedirect. Literature published before October 2025 was retrieved, and a combined search strategy was employed to obtain original studies reporting phospholipid content in human milk. The following search terms were used for English databases: (“phospholipid” OR “phosphatidylcholine” OR “phosphatidylethanolamine” OR “phosphatidylinositol” OR “phosphatidylserine” OR “sphingomyelin”) AND (“breast milk”OR “human milk”OR “mother milk”OR “woman milk”OR “women milk” OR “lactating milk”). For Chinses databases, we optimized the searching strategy to (“human milk” OR “breast milk”) AND (“phospholipid”). This systematic review and meta-analysis followed the PRISMA 2020 guidelines and was registered at PROSPERO (https://www.crd.york.ac.uk/PROSPERO/view/CRD420261445346). The PRISMA checklist of items in this systematic review and meta-analysis can be found in Supplementary Table 1.

### 2.2 Literature inclusion and exclusion criteria

We adopted as inclusion criteria the studies that:

- Publications in Chinese or English.
- Observational studies (cross-sectional or cohort study) or intervention studies with a control group not receiving any intervention.
- Mothers were aged between 18 and 45 years.

The studies were excluded that:

- Studies using mixed breast milk or with unspecified lactation stage.
- Duplicate publications.
- Mothers with any medical conditions or diseases.
- Systematic reviews and conference abstracts/papers.
- Studies with incomplete or unavailable data.
- Studies involving dietary interventions.

### 2.3 Article screening and data abstraction

Through a preliminary literature search, duplicate and irrelevant studies were excluded based on titles and abstracts. The remaining articles were read in full and included according to the predefined inclusion and exclusion criteria. This process was conducted independently by two researchers. Any disagreements regarding eligibility were resolved through discussion and consultation with a third professional.

The following information was extracted from the final included articles, which included the first author, year of publication, country, lactation stage, sample size, detection method, relevant characteristics of mother (age, gestational weeks etc.) and data of phospholipids. Phospholipids include SM, PC, PE, PS, PI, and total phospholipids. Current studies have quantified both the concentration and relative proportions of major phospholipid classes, therefore, both types of data were extracted.

### 2.4 Assessment of study quality

We used the Agency for Healthcare Research and Quality (AHRQ) guideline for methodological quality assessment of the included literature (15). AHRQ scale consists 8-item instrument with a yes/no/unclear response option: the “Yes” should be obtained score “1,”“No” or “unclear” was scored “0.” Articles were scored as follows: 0–2 means low quality; 3–5 means moderate quality; 6–8 means high quality.

### 2.5 Data conversion

Phospholipid concentration reported in different units was uniformly converted to mg/L. For instance, millimolar values were converted to milligrams by multiplication with the corresponding molecular weight. The molecular weights of the five phospholipid subclasses are as follows: SM 754.4, PE 737.6, PC 758.4, PI 864.4 and PS 787.1 (16). In addition, values reported per kilogram of human milk were converted to per liter by multiplying by 1.032. According to the Wan et al. (17), when data was reported as mean ± SEM, mean (95% CI) or as median (IQR), we standardized to the same units (mean ± SD) for further analysis. Given the varying lactation stages reported across publications, lactation stages were classified as follows: 0–6 days (colostrum); 7–14 days (transitional milk); 15–365 days (mature milk). If multiple data existed in the same lactation stage within one study, the weighted mean and standard deviation were calculated using the weighting method. The calculation formulas were as follows, where the individual mean value, sample size and standard deviation of individual research are represented by $M_{i}$ , $n_{i}$ and $S_{i}$ , respectively.

$$\text{Means}=\frac{\left(n_{1}\times M_{1}+n_{2}\times M_{2}+n_{3}\times M_{3}+\cdots +n_{i}\times M_{i}\right)}{\left(n_{1}+n_{2}+n_{3}+\cdots +n_{i}\right)}$$

$$A_{i}=S_{i}^{2}(n_{i}-1)+M_{i}^{2}\times n_{i}$$

$$\mathrm{SDs}=\sqrt{\frac{\sum A_{i}-\frac{{\left[\sum (M_{i}n_{i})\right]}^{2}}{N}}{N-1}}$$

A weighted mean and standard deviation were determined for a common lactation stage. If the result form an individual study exceeded the range of Mean ± 2 SD, the data was considered an outlier and was excluded. The proportion of each phospholipid subclass was calculated as the concentration of the respective subclass divided by the total phospholipid concentration.

### 2.6 Statistical analysis

The meta-analysis was performed using SPSS AU software. The “mean meta-analysis” was used to pool phospholipid concentrations as the mean and 95% confidence interval (95% CI). Heterogeneity was evaluated using the *I*^2^ statistic and Cochran’s *Q* test. *I*^2^ > 50% indicated significant heterogeneity across studies, and the random-effects model was applied; otherwise, the fixed-effects model was used. The results were presented in forest plots. Individual trials were examined using sensitivity analysis at each stage of lactation. We used Egger’s test to evaluate the publication bias of the study, and *p* < 0.05 suggested that there was publication bias. Meanwhile, we conducted subgroup analysis for mature milk. The subgroup analyses were performed stratified by national economic level (developed countries and developing countries), geographical location (China and non-China) and detection methods (^31^P NMR, HPLC-ELSD, LC–MS and other).

## 3 Results

### 3.1 Study selection

After removing duplicates, 2013 articles remained, and 1963 articles were excluded by reviewing titles and abstracts. According to the inclusion and exclusion criteria, 25 studies were finally included after full-text reading (11 proportion, 18 concentration, and 4 of which reported both), and the screening process is shown in Figure 1. Of these included studies, 18 focusing on phospholipid concentrations were synthesized for meta-analysis.

**Figure 1:**
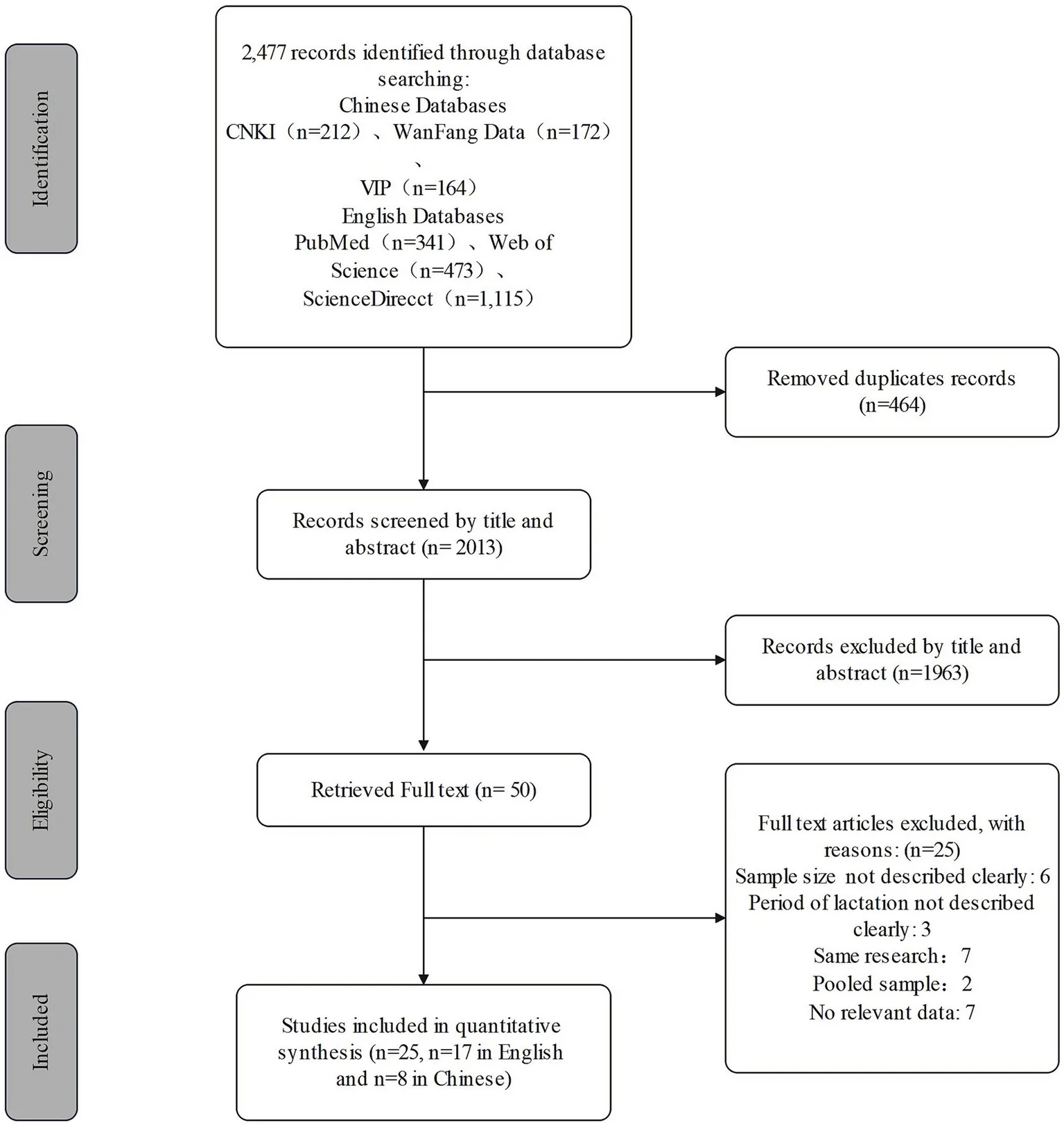
Flow diagram of literature review.

### 3.2 Study characteristics

These studies varied in sample size, lactation stage, detection method, and geographical region. The basic characteristics of all included studies are summarized in Table 1. Among them, one study (18) reported phospholipid concentrations in breast milk from mothers of both preterm and term infants; however, only data from mothers of healthy term infants were used in the present study. The most common detection methods used for phospholipid quantification included high performance liquid chromatography coupled with evaporative light-scattering detector (HPLC-ELSD), liquid chromatography–tandem mass spectrometry (LC–MS), ^31^P-nuclear magnetic resonance spectroscopy (^31^P-NMR), thin-layer chromatography (TLC), and others.

**Table 1:** Summary characteristics of included studies.

References	Sample	Milk type	Region	Country	Age of mothers (year)	Gestational age (week)	Reporting unit	Detection method
Wang et al., 2000 (22)	20	CM	Asia	Japan	N.S	37–40	Proportion	TLC
Ilcol et al., 2005 (21)	153	MM	Asia	Turkey	N.S	N.S	Concentration	TLC
Sala-Vila et al., 2005 (30)	66	CM, TM, MM	Europe	Spain	17–36	38–42	Proportion	HPLC-ELSD
Shoji et al., 2005 (29)	44	CM, TM, MM	Asia	Japan	18.5–35.8	38–40.6	Proportion	TLC
Benoit et al., 2010 (31)	4	MM	Europe	France	33 ± 3	N.S	Proportion	TLC
Lopez et al., 2011 (4)	3	MM	Europe	France	N.S	N.S	Proportion	HPLC-ELSD
Garcia et al., 2012 (32)	22	MM	Europe	France	N.S	N.S	Proportion concentration	^31^P NMR
Giuffrida et al., 2013 (34)	50	MM	Asia	Singapore	31.1 ± 3.1	N.S	Concentration	^31^P NMR
Thakkar et al., 2013 (35)	150	MM	Asia	Singapore	31.1 ± 3.1	N.S	Concentration	HPLC-ELSD
Xia, 2015 (23)	309	CM, TM, MM	Asia	China	27.86 ± 3.22	39.28 ± 0.68	Proportion	ACQUITY UPLC
Tavazzi et al., 2017 (47)	15	MM	Asia	Singapore	31.1 ± 3.1	N.S	Concentration	HPLC–MS/MS
Ma et al., 2017 (33)	168	CM, TM, MM	Asia	Malaysia	19–40	N.S	Concentration	HPLC-ESI-MS
McJarrow et al., 2019 (16)	81	TM, MM	Asia	United Arab Emirates	19–40	27–42	Concentration	HPLC–MS
Wei et al., 2019 (18)	30	CM, TM, MM	Asia	China	28.2 ± 3.6	39.2 ± 0.7	Concentration	^31^P NMR
Wu et al., 2019 (24)	248	CM, TM, MM	Asia	China	30 ± 3	39 ± 1	Proportion concentration	HPLC-UV
Fong et al., 2020 (48)	140	MM	Asia	China	N.S	N.S	Concentration	HPLC–MS
Zhao et al., 2020 (49)	183	CM, TM, MM	Asia	China	21.9–36.3	N.S	Concentration	UPLC/Q-TOF-MS
Zhou, 2020 (25)	160	MM	Asia	China	N.S	N.S	Proportion concentration	HILIC-ESI-MS
Duan et al., 2021 (50)	33	MM	Asia	Korea	23–41	≥35	Proportion concentration	^31^P NMR
Liang et al., 2021 (51)	646	MM	Asia	China	24.6–33.9	N.S	Concentration	HPLC-ELSD
Weng, 2021 (26)	9	MM	Asia	China	N.S	N.S	Concentration	^31^P NMR
Lin et al., 2022 (52)	101	CM, TM, MM	Asia	China	N.S	N.S	Concentration	^31^P NMR
Yang et al., 2022 (53)	2,270	CM, TM, MM	Asia	China	21–44	37–42	Concentration	HPLC-ELSD
Li, 2023 (54)	90	CM, TM, MM	Asia	China	N.S	N.S	Concentration	UPSFC-Q-TOF-MS
Zhu et al., 2023 (27)	201	MM	Asia	China	29.2–41.3	N.S	Proportion	LC–MS/MS

CM, colostrum milk; TM, transitional milk; MM, mature milk. N.S, not specified. TLC: thin-layer chromatography; HPLC-ELSD: high performance liquid chromatography coupled with evaporative light-scattering detector; ^31^P-NMR: ^31^P-nuclear magnetic resonance spectroscopy; HPLC–MS/MS:high performance liquid chromatography coupled with tandem mass spectrometry; HPLC-ESI-MS:high performance liquid chromatography coupled with electrospray ionization mass spectrometry; HPLC-UV:high performance liquid chromatography coupled with ultraviolet detector; UPLC/Q-TOF-MS: ultra-performance liquid chromatography/quadrupole-time-of-flight mass spectrometry; HILIC-ESI-MS: hydrophilic interaction liquid chromatography coupled with electrospray ionization tandem mass spectrometry; UPSFC-Q-TOF-MS:ultra-high performance supercritical fluid chromatography tandem quadrupole time-of-flight mass spectrometry.

Supplementary Table S3 summarizes the methodological quality assessment of the included studies: 18 studies were graded as high quality, 7 studies were as moderate quality.

### 3.3 Total phospholipid concentration in human milk

The meta-analysis of total phospholipid concentration is shown in Figure 2. A random-effects model was used to perform the meta-analysis of total phospholipid concentrations in human milk across different lactation stages. The pooled total phospholipid concentration with 95% CI in colostrum, transitional milk, and mature milk were 251.60 mg/L (175.62, 327.58), 234.71 mg/L (171.07, 298.35), and 197.00 mg/L (166.08, 227.93), respectively. The overall pooled concentration across all lactation stages was 212.79 mg/L (95% CI: 188.81–236.77), with significant overall heterogeneity (*I*^2^ = 99.33%, *p* = 0.00). Although a decreasing trend in the total phospholipid concentration was observed from colostrum to transitional milk to mature milk (251.60 vs. 234.71 vs.197.00), the between-group difference was not statistically significant (*p* = 0.16).

**Figure 2:**
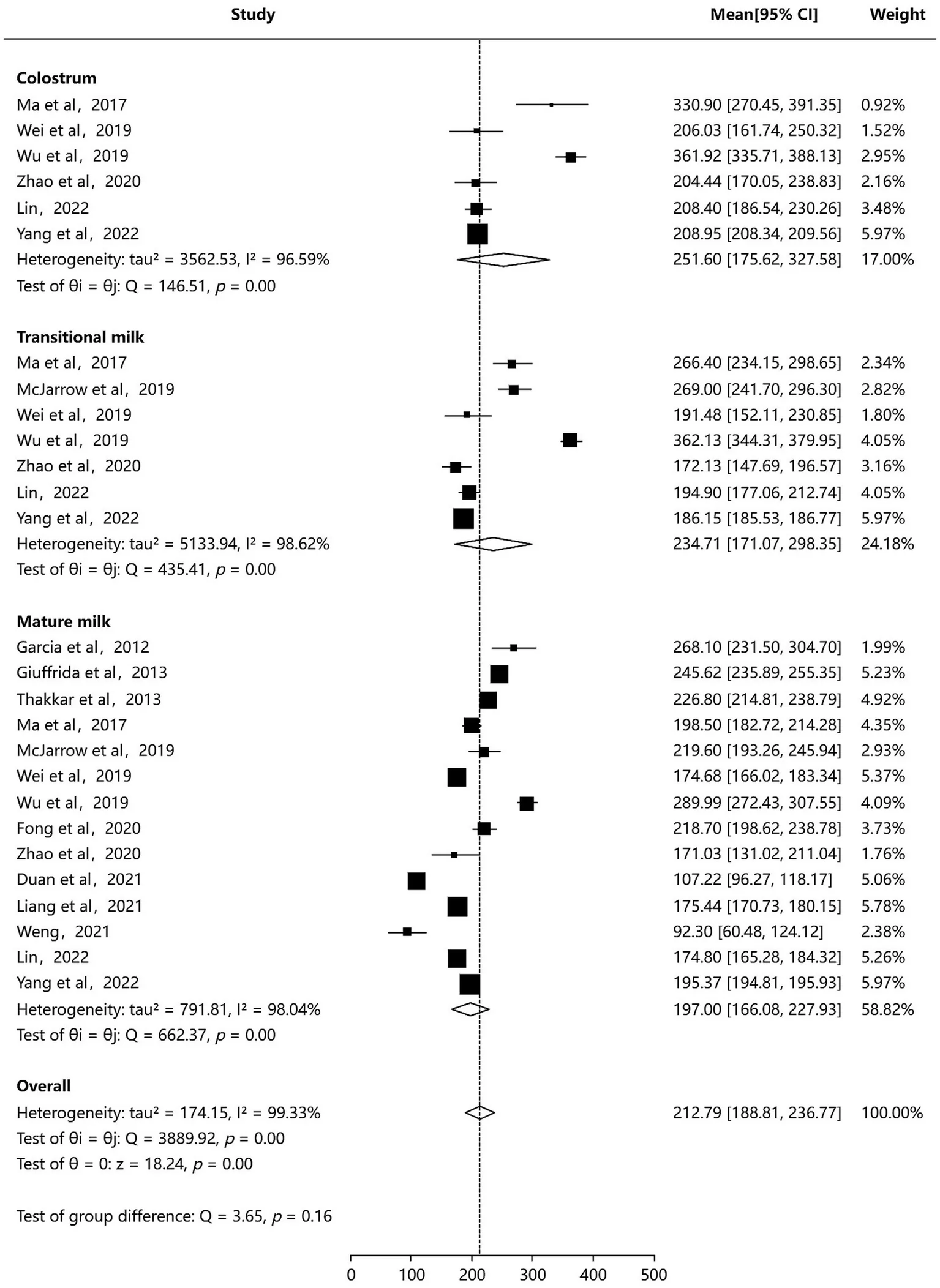
Forest plot of the meta-analysis on total phospholipid concentration. Squares represent the weighted mean difference for each study, with the size of each square reflecting the study-specific weight. The 95% confidence interval is indicated by the horizontal bars, and the diamond represents the pooled weighted mean difference.

### 3.4 The concentration of phospholipid subclasses in human milk

Table 2 summarizes the changes in the concentrations of phospholipid subclasses in human milk at different lactation stages. The concentrations of the five phospholipid subclasses throughout the entire lactation period were as follows: SM 66.50 mg/L (95% CI: 58.47–74.52), PE 57.74 mg/L (95% CI: 52.21–63.26), PC 52.20 mg/L (95% CI: 43.47–60.93), PS 13.18 mg/L (95% CI: 10.03–16.34) and PI 10.34 mg/L (95% CI: 8.23–12.45). The pooled concentration of PC in colostrum was 64.72 mg/L (95% CI: 44.56–84.88), decreased to 50.60 mg/L (95% CI: 42.89–58.32) in transitional milk, and further stabilizes at 50.09 mg/L (95% CI: 34.59–69.59) in mature milk. The concentration of PC decreased significantly from colostrum to transitional milk, with an overall reduction of 21.82%. Nevertheless, longitudinal changes of PC over lactation was not significant according to subgroup heterogeneity analysis using a random effect model (*p* = 0.27). The pooled concentrations of PI were 10.55 mg/L, 10.32 mg/L and 10.25 mg/L in colostrum, transitional milk and mature milk, respectively. The concentration of PI showed no significant fluctuation across different lactation stages, suggesting that PI maintains a relatively stable level during the lactation process. Overall, the pooled levels of SM, PE, PS and PI remained relatively stable across the three lactation stages, with non-significant differences among colostrum, transitional milk and mature milk (*p* > 0.05).

**Table 2:** Meta-analysis of phospholipid subclass concentration.

Phospholipid class	Colostrum		Transitional milk		Mature milk		*p*	*I* ^2^
	Sample	Mean (95%CI)	Sample	Mean (95%CI)	Sample	Mean (95%CI)		
Total	443	251.60 (175.62, 327.58)	496	234.71 (171.07, 298.35)	3,486	197.00 (166.08, 227.93)	0.16	99.32
SM	494	63.78 (41.65, 85.92)	527	63.89 (40.80, 86.98)	3,498	70.54 (60.67, 80.42)	0.71	99.64
PE	473	58.83 (48.07, 69.60)	527	57.23 (49.22, 65.24)	3,366	57.21 (47.75, 66.67)	0.95	99.95
PC	424	64.72 (44.56, 84.88)	431	50.60 (42.89, 58.32)	3,338	50.09 (34.59, 69.59)	0.27	99.85
PS	318	13.67 (−0.18, 27.52)	358	14.21 (3.60, 24.82)	3,015	12.60 (8.61, 16.60)	0.92	99.96
PI	403	10.55 (3.32, 17.78)	401	10.32 (5.11, 15.53)	3,212	10.25 (7.53, 12.96)	1.00	99.87

All analytes are reported in mg/L; *p* values for between-group differences in lactation stages.

Throughout the all lactation process, PC and SM are the two most abundant, followed by PE and then PS and PI (Figure 3). Most of analytical results presented elevated *I*^2^ values exceeding 99.0%, which demonstrated significant heterogeneity among individual studies. SM is essential for myelin sheath formation of nerve fibers in the central and peripheral nervous systems, which may influence the structure and organization of brain regions, neurogenesis and synaptogenesis (19). As the primary source of choline for neonates, PC is critical for brain development. PC is metabolized by phospholipase D present in synaptic membranes into phosphatidic acid and choline. Choline is rapidly taken up by cholinergic neurons, then combines with acetyl-coenzyme A to form acetylcholine in brain tissue. Acetylcholine, which is associated with motor function and memory, accelerates signal transmission between brain nerve cells and significantly enhances brain activity (20, 21). The proportion of long-chain polyunsaturated fatty acids in PE accounts for a remarkably high 59% of total fatty acids, especially unsaturated fatty acids such as arachidonic acid (ARA) and docosahexaenoic acid (DHA) (22). Thus, PE serves as an excellent source of ARA and DHA, and these nutrients can significantly promote brain and visual development in infants. Therefore, SM, PC, and PE in human milk are of great importance for infant growth and development.

**Figure 3:**
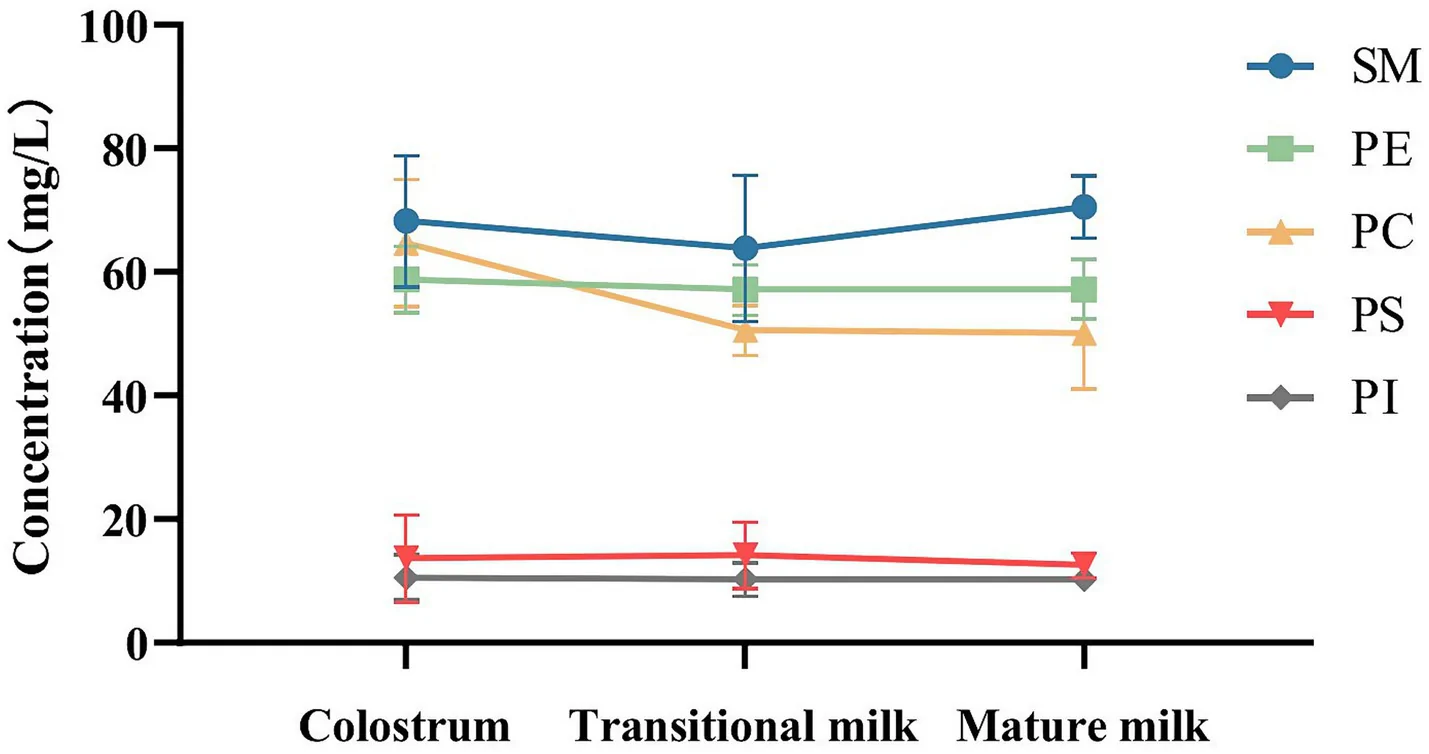
Overview of five phospholipid subclasses during lactation.

### 3.5 Sensitivity analysis

Sensitivity analysis was performed using the leave-one-out method, where the pooled effect size was recalculated after sequentially excluding each individual study. All analysis revealed substantial heterogeneity (*I*^2^ in 66–100%) (Supplementary Tables S4–21). No significant changes were observed in the combined effect of total phospholipids and phospholipid subclass concentrations across the three lactation stages, suggesting that none of the included single studies dominated the pooled estimates. Nevertheless, given the extremely high heterogeneity across studies, the credibility of these pooled results remains limited.

### 3.6 Publication bias

Egger’s test revealed evidence of publication bias for SM in mature milk (*p* = 0.018). After reanalysis using the trim-and-fill method, the pooled effect size of SM in mature milk was 55.61 mg/L (95% CI: 43.10–68.11) (Figure 4), which was lower than the original pooled effect estimate (Table 2).

**Figure 4:**
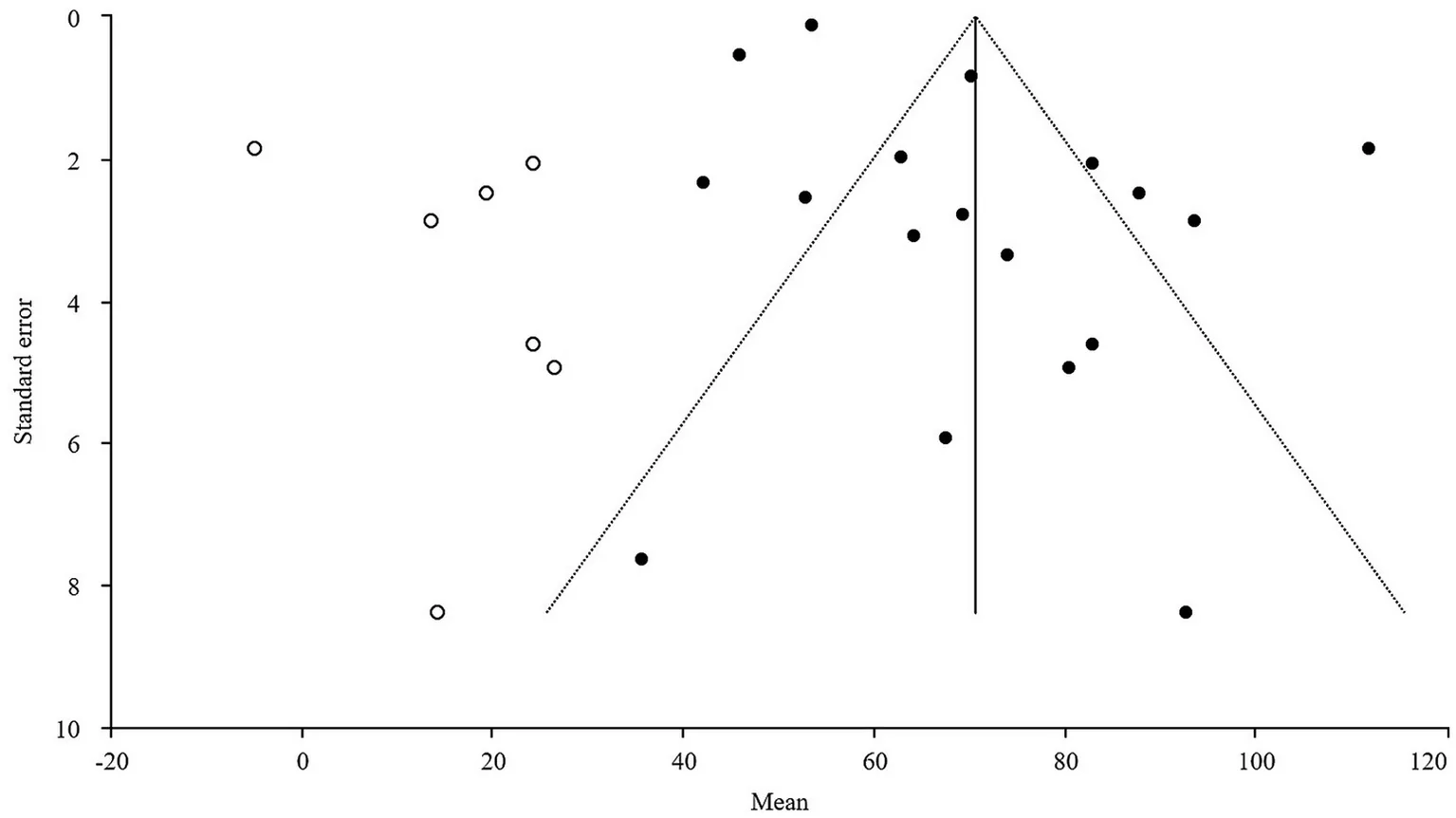
Trim-and-fill analysis estimated the number of possible missing studies for the SM concentration in mature milk.

### 3.7 Subgroup analysis

Additional subgroup analysis of mature milk were performed by economic levels of countries, geographical location and detection methods. Table 3 shows the total phospholipid concentrations estimated by subgroup analysis. By economic level, the concentration was 211.19 mg/L (95%CI: 96.77–325.61) in developed countries, and 197.00 mg/L (95% CI: 166.08–227.93) in developing countries, with no significant difference between subgroups (*p* = 0.65). By geographical location, the concentration was 189.80 mg/L (95%CI: 146.11–233.49) for China versus 210.26 mg/L (95% CI: 151.33–269.20) for non-China regions, and the between subgroup difference was not statistically significant (*p* = 0.49). Subgroup analysis based on detection methods revealed a significant between group difference (*p* < 0.001). The pooled concentration was 176.70 mg/L (95% CI: 103.37–250.03) for the ^31^P NMR subgroup, 198.67 mg/L (95% CI: 135.01–261.13) for the HPLC-ELSD subgroup, 205.56 mg/L (95% CI: 175.29–235.82) for the LC–MS subgroup, and 289.99 mg/L (95% CI: 272.43–307.55) for the other detection method. Moderate heterogeneity (*I*^2^ = 52.85%) was found in the LC–MS subgroup, while extremely high within subgroup heterogeneity was observed in the remaining subgroups. Additionally, significant between-subgroup differences attributable to detection methods were identified for PE and PI (Supplementary Tables S22–26). The results indicated that economic level and geographical region did not produce statistically significant between subgroup differences and could not explain the high heterogeneity observed in this meta-analysis. High within-subgroup heterogeneity still remained in most stratification, suggesting that other confounding variables such as maternal diet and gestational age may contribute to heterogeneity.

**Table 3:** Subgroup analysis of total phospholipids in mature milk.

Subgroup variable	Subgroup	Mean (95%CI)	Heterogeneity *I*^2^	*p* value
Economic level	Developed	211.19 (96.77, 325.61)	99.24	0.65
	Developing	197.00 (166.08, 227.93)	96.66	
Geographical location	China	189.80 (146.11, 233.49)	97.37	0.49
	Non-China	210.26 (151.33, 269.20)	98.73	
Detection methods	^31^P NMR	176.70 (103.37, 250.03)	98.73	0.00
	HPLC-ELSD	198.67 (135.01, 261.13)	97.89	
	LC–MS	205.56 (175.29, 235.82)	52.85	
	Other	289.99 (272.43, 307.55)	–	

LC–MS methods included HPLC-MS, HPLC-MS/MS, HPLC-ESI-MS, HILIC-ESI-MS, UPLC/Q-TOF-MS and LC-MS/MS. Other methods included TLC, HPLC-UV and UPSFC-Q-TOF-MS.

### 3.8 The proportion of phospholipid subclass in human milk

Furthermore, the relative proportions of individual phospholipid classes in total phospholipids were analyzed. Figure 5 depicts the regional variations in the proportions of five phospholipid subclasses across China (23–27), the United States (28), Japan (22, 29), Spain (30), France (4, 31, 32), the United Arab Emirates (16), Malaysia (33), and Singapore (34, 35). PC is the dominant component in China (37.5%), while SM prevails in Spain (40.3%), the UAE (35.8%), and Singapore (36.3%). Japan and Southeast Asian countries (Malaysia, Singapore) contain high PE, which peaks at 33.6% in Japan. Across all regions, PI uniformly constitutes the least abundant phospholipid subclass, with proportions consistently below 6%, whereas PS maintains intermediate, regionally variable levels ranging from 3.7% (America) to 14.8% (Malaysia). Across different countries, PC, SM and PE are the dominant phospholipid subclasses in human milk. We found that the proportion of PE from Spanish mothers was lower than in other countries. This difference may be attributed to the fact that some studies included measurements of lysophosphatidylethanolamine within the PE total, whereas the Sala-Vila et al. (30) excluded this result. The phospholipid content in breast milk varies among mothers from different countries, possibly due to different dietary habits. For example, Japanese mothers have long had a diet rich in seafood, so their breast milk might contain higher levels of PE, because deep-sea fish and shrimp are rich in various unsaturated fatty acids that make up this type of phospholipid. In conclusion, these geographic divergences in phospholipid subclass abundance may reflect disparities in regional dietary patterns or population physiological characteristics.

**Figure 5:**
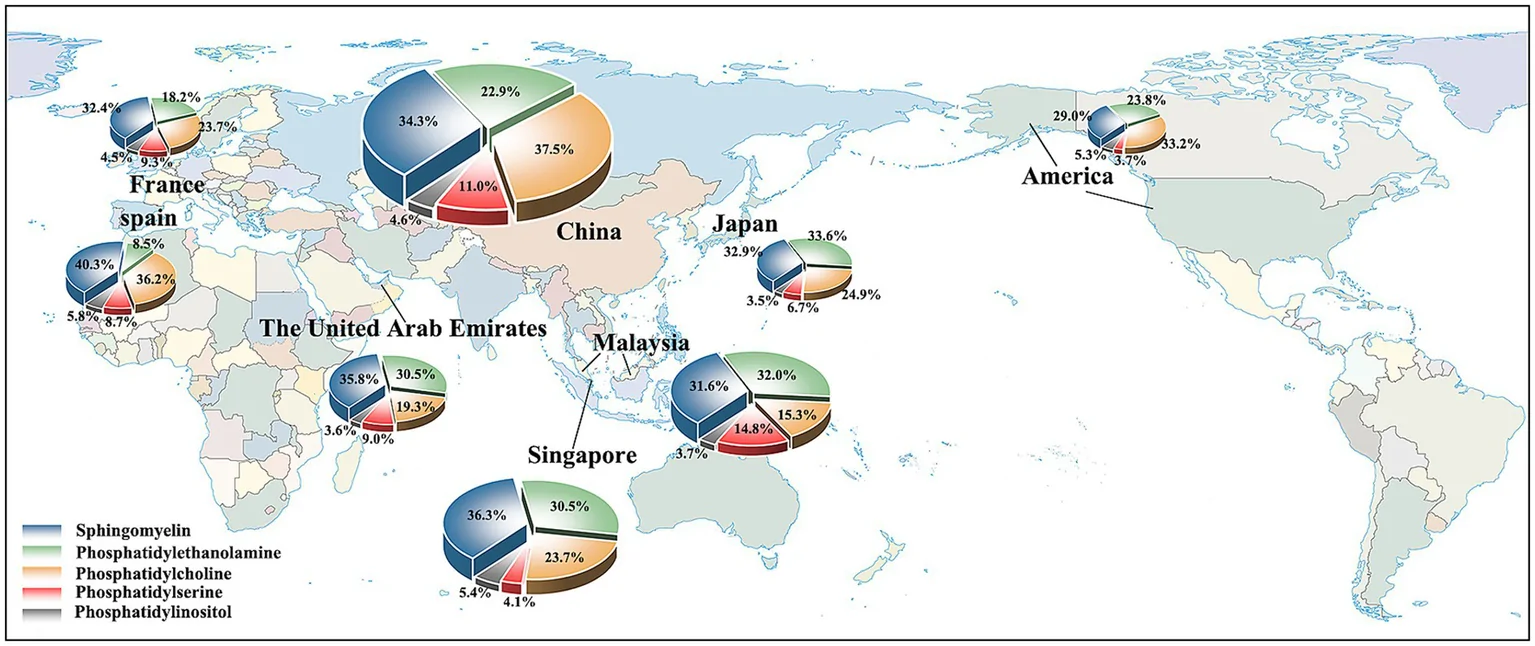
Relative proportions of phospholipid subclasses in human milk.

## 4 Discussion

### 4.1 Changes of total phospholipids and phospholipid subclasses in human milk during lactation and their influencing factors

Phospholipids are important components of breast milk and play a crucial role in promoting infant cognitive development, accelerating intestinal maturation, and regulating cholesterol metabolism. Through a literature search, this study included 25 articles and summarized the concentrations of total phospholipids and phospholipid subclasses in breast milk worldwide. The results indicate that the concentration of total phospholipids changes dynamically during lactation. As the lactation period extends, the total phospholipid concentration decreases. The total phospholipid content is higher in early lactation, which may be associated with maternal physical status of mothers and the physiological developmental needs of infants. During the colostrum stage, mammary gland cells exhibit strong secretory activity, resulting to a large number of secreted milk fat globules and a high level of total phospholipids (36, 37). Moreover, the size of milk fat globules has been shown to be negatively correlated with total phospholipid content (38, 39), which suggests that the higher concentration of phospholipids in colostrum can better disperse milk fat globules and expand the contact area of lipase during gastrointestinal digestion.

In addition, we quantified the concentrations of five phospholipid subclasses and characterized their overall dynamic trends throughout lactation (Figure 3). PC showed a decreasing trend from colostrum to transitional milk, with its concentration declining from 64.72 mg/L to 50.60 mg/L. In contrast, SM, PE, PS and PI remained stable across different lactation stages without any significant fluctuations. We observed that SM and PC constituted the major PLs in human milk across all lactation stages, which is consistent with previous investigations (40–42). The abundant phospholipid fractions in colostrum, especially PC and SM, act as transport carriers for critical fatty acids including DHA and ARA and may supply essential substrates to support neonatal neurodevelopment. Although PS and PI together account for a relatively small proportion of human milk phospholipids, PS can activate signaling pathways such as protein kinase C, thereby affecting neuronal survival and synaptic plasticity. PI can bind to specific proteins and participate in cell recognition and signal transduction of growth factors. Both play indispensable roles in maintaining cell membrane fluidity and mediating cellular signal transmission.

Phospholipid concentrations in human milk exhibits significant variability among different populations, with such variations are influenced by factors including gestational age, lactation stage, maternal diet, and region. Lindahl et al. (43) analyzed the phospholipid composition of human milk from mothers of preterm and term infants at different lactation stages. The results showed that the concentrations of PC, SM and PL in breast milk from preterm mothers were significantly higher than those from term mothers throughout the lactation (*p* < 0.05). As lactation progresses, the total PLs concentration in human milk of both preterm and term infants decreases significantly, and the difference in total PL concentration between the two groups gradually decreasing. The abundance of phospholipid subclasses in human milk during different lactation stages also changes dynamically. Song et al. (44) reported that the two major PLs in colostrum (0–5 days) and transitional milk (10–15 days) were PS and PE. In contrast, SM emerged as the second most abundant PL in early mature milk (40–45 days), late mature milk (200–240 days), and long-term mature milk (300–400 days). Liu et al. (45) compared the composition and content of PLs in breast milk from Tibet and other regions of China, including Beijing, Luoyang, Liuyang and Tangshan. They found that the concentrations of PC, PE, and SM in breast milk from Tibet were significantly lower than those in other regions. The daily intake and consumption frequency of eggs rich in PLs were lower in the Tibet region than in other regions. Moreover, PLs-rich foods such as fish, shrimp, legumes and nuts were also rarely consumed. This may be the main reason for the significant difference in PLs content in human milk between Tibet and other regions. Claumarchirant et al. (46) quantified the phospholipid concentrations in colostrum, transitional milk, and mature milk of mothers from two different geographical regions (central and coastal) in Spain. The results showed that the levels of minor phospholipids (PI, PS, and PC) were higher in the coastal area, while no significant differences were observed in the levels of PE and SM between the two regions. Furthermore, Thakkar et al. (35) reported that infant sex may affect the PLs composition of human milk. Mothers with male infants had higher concentrations of SM, PC, PE, and PI in their human milk than those with female infants. This difference may be attributed to the distinct energy requirements for individual growth and development.

### 4.2 Study limitation

This meta-analysis provides a global overview of total and subclass phospholipid concentrations across three lactation stages, yet the extremely high heterogeneity fundamentally limits the generalizability and robustness of the overall pooled estimates. As a result, the meta results for total and subclass phospholipid concentrations should be interpreted with caution. We performed subgroup analysis, leave-one-out sensitivity analysis, Egger’s test, and trim-and-fill tests to explore the substantial between-study heterogeneity. However, these analysis failed to fully account for heterogeneity, and high heterogeneity persisted across most comparisons. In the subgroup analysis, these moderators (national economic level, geographical location and detection methods) only partially explained variance in a limited number of phospholipid fractions (total phospholipids, PE, and PI in mature milk), whereas no significant subgroup differences were observed for SM, PC, and PS to resolve their marked inter-study discrepancies. This implies that other potential influencing factors such as gestational age and lactation stage may exert effects on human milk phospholipid profiles. But due to incomplete documentation of these factors across most included studies, we could not perform corresponding subgroup analyses. In addition, maternal dietary intake, differences in sampling protocols (foremilk, hindmilk, mixed whole milk) and the human milk pre-extraction treatments, that were incompletely documented in most studies are also important unmeasured confounders. These variables were incompletely reported in the majority of the included studies, further limit the comprehensive exploration of the sources of between-study heterogeneity, which represents a key limitation of this study. Our subgroup analysis was restricted exclusively to mature milk due to limited stratified data available for colostrum and transitional milk, creating an incomplete understanding of how moderators shape phospholipid levels in early lactation. In the future, large-scale and standardized multi-regional cohort studies are urgently required to uniformly record maternal dietary status, sampling modes and lipid pretreatment procedures, which can quantify the unmeasured confounders identified herein.

## 5 Conclusion

This study analyzed the concentrations of total phospholipids and five phospholipid subclasses reported in 25 articles published from 2000 to 2025. The overall trend indicated that phospholipids were abundant in early breast milk and gradually declined as lactation progressed, reaching the lowest concentration in mature milk. Among the five subclasses, SM, PC and PE were the predominant components, followed by PI and PS. The dynamic changes of phospholipids during lactation correspond to the growth and developmental needs of infants, providing a reference for the formulation design of phospholipid content in infant formula.

## Data Availability

The original contributions presented in the study are included in the article/Supplementary material, further inquiries can be directed to the corresponding author.
