# Supplementary material for: A systematic review and meta-analysis of phospholipids in human milk
Source: Front Nutr. 2026 Sep 23;13:1929517. doi: 10.3389/fnut.2026.1929517 (PMC13642143; doi:10.3389/fnut.2026.1929517)
Supplement: Supplementary file 1 [file Supplementary_file_1.docx]

Supplementary Material

**Supplementary Figure 1.** Forest plot of concentrations of five phospholipid subclasses, in the following order: SM, PE, PC, PS and PI.

**Supplementary Table 1.** PRISMA checklist

| **Topic** | **No.** | **Item** | **Location where item is reported** |
| --- | --- | --- | --- |
| **TITLE** |  |  |  |
| **Title** | 1 | Identify the report as a systematic review. | Title |
| **ABSTRACT** |  |  |  |
| **Abstract** | 2 | See the PRISMA 2020 for Abstracts checklist |  |
| **INTRODUCTION** |  |  |  |
| **Rationale** | 3 | Describe the rationale for the review in the context of existing knowledge. | Introduction Paragraph 1-2 |
| **Objectives** | 4 | Provide an explicit statement of the objective(s) or question(s) the review addresses. | Introduction Paragraph 3 |
| **METHODS** |  |  |  |
| **Eligibility criteria** | 5 | Specify the inclusion and exclusion criteria for the review and how studies were grouped for the syntheses. | Section 2.2 |
| **Information sources** | 6 | Specify all databases, registers, websites, organisations, reference lists and other sources searched or consulted to identify studies. Specify the date when each source was last searched or consulted. | Section 2.1 |
| **Search strategy** | 7 | Present the full search strategies for all databases, registers and websites, including any filters and limits used. | Supplementary Table 2 |
| **Selection process** | 8 | Specify the methods used to decide whether a study met the inclusion criteria of the review, including how many reviewers screened each record and each report retrieved, whether they worked independently, and if applicable, details of automation tools used in the process. | Section 2.3 |
| **Data collection process** | 9 | Specify the methods used to collect data from reports, including how many reviewers collected data from each report, whether they worked independently, any processes for obtaining or confirming data from study investigators, and if applicable, details of automation tools used in the process. | Section 2.3 |
| **Data items** | 10a | List and define all outcomes for which data were sought. Specify whether all results that were compatible with each outcome domain in each study were sought (e.g. for all measures, time points, analyses), and if not, the methods used to decide which results to collect. | Section 2.3 |
|  | 10b | List and define all other variables for which data were sought (e.g. participant and intervention characteristics, funding sources). Describe any assumptions made about any missing or unclear information. | Table 1 |
| **Study risk of bias assessment** | 11 | Specify the methods used to assess risk of bias in the included studies, including details of the tool(s) used, how many reviewers assessed each study and whether they worked independently, and if applicable, details of automation tools used in the process. | Section 2.4 |
| **Effect measures** | 12 | Specify for each outcome the effect measure(s) (e.g. risk ratio, mean difference) used in the synthesis or presentation of results. | Section 2.5 |
| **Synthesis methods** | 13a | Describe the processes used to decide which studies were eligible for each synthesis (e.g. tabulating the study intervention characteristics and comparing against the planned groups for each synthesis (item 5)). | Section 2.5 |
|  | 13b | Describe any methods required to prepare the data for presentation or synthesis, such as handling of missing summary statistics, or data conversions. | Section 2.5 |
|  | 13c | Describe any methods used to tabulate or visually display results of individual studies and syntheses. | Section 2.6 |
|  | 13d | Describe any methods used to synthesize results and provide a rationale for the choice(s). If meta-analysis was performed, describe the model(s), method(s) to identify the presence and extent of statistical heterogeneity, and software package(s) used. | Section 2.6 |
|  | 13e | Describe any methods used to explore possible causes of heterogeneity among study results (e.g. subgroup analysis, meta-regression). | Section 2.6 |
|  | 13f | Describe any sensitivity analyses conducted to assess robustness of the synthesized results. | Section 2.6 |
| **Reporting bias assessment** | 14 | Describe any methods used to assess risk of bias due to missing results in a synthesis (arising from reporting biases). | NA |
| **Certainty assessment** | 15 | Describe any methods used to assess certainty (or confidence) in the body of evidence for an outcome. | NA |
| **RESULTS** |  |  |  |
| **Study selection** | 16a | Describe the results of the search and selection process, from the number of records identified in the search to the number of studies included in the review, ideally using a flow diagram. | Figure 1 |
|  | 16b | Cite studies that might appear to meet the inclusion criteria, but which were excluded, and explain why they were excluded. | NA |
| **Study characteristics** | 17 | Cite each included study and present its characteristics. | Section 3.2 |
| **Risk of bias in studies** | 18 | Present assessments of risk of bias for each included study. | Supplementary Table 3 |
| **Results of individual studies** | 19 | For all outcomes, present, for each study: (a) summary statistics for each group (where appropriate) and (b) an effect estimate and its precision (e.g. confidence/credible interval), ideally using structured tables or plots. | Figure 2，Supplementary Figure 1 |
| **Results of syntheses** | 20a | For each synthesis, briefly summarise the characteristics and risk of bias among contributing studies. | Section 3.3、3.4 |
|  | 20b | Present results of all statistical syntheses conducted. If meta-analysis was done, present for each the summary estimate and its precision (e.g. confidence/credible interval) and measures of statistical heterogeneity. If comparing groups, describe the direction of the effect. | Section 3.3、3.4, Table3，Supplementary Table 22-26 |
|  | 20c | Present results of all investigations of possible causes of heterogeneity among study results. | Figure 2，Supplementary Figure 1 |
|  | 20d | Present results of all sensitivity analyses conducted to assess the robustness of the synthesized results. | Section 3.5, Supplementary Table 4-21 |
| **Reporting biases** | 21 | Present assessments of risk of bias due to missing results (arising from reporting biases) for each synthesis assessed. | NA |
| **Certainty of evidence** | 22 | Present assessments of certainty (or confidence) in the body of evidence for each outcome assessed. | Section 3.3、3.4 |
| **DISCUSSION** |  |  |  |
| **Discussion** | 23a | Provide a general interpretation of the results in the context of other evidence. | Section 4 |
|  | 23b | Discuss any limitations of the evidence included in the review. | Section 4 |
|  | 23c | Discuss any limitations of the review processes used. | Section 4.2 |
|  | 23d | Discuss implications of the results for practice, policy, and future research. | Section 4.2 |
| **OTHER INFORMATION** |  |  |  |
| **Registration and protocol** | 24a | Provide registration information for the review, including register name and registration number, or state that the review was not registered. | Section 2.1 |
|  | 24b | Indicate where the review protocol can be accessed, or state that a protocol was not prepared. | Section 2.1 |
|  | 24c | Describe and explain any amendments to information provided at registration or in the protocol. | Section 2.1 |
| **Support** | 25 | Describe sources of financial or non-financial support for the review, and the role of the funders or sponsors in the review. | Funding |
| **Competing interests** | 26 | Declare any competing interests of review authors. | Conflict of Interest |
| **Availability of data, code and other materials** | 27 | Report which of the following are publicly available and where they can be found: template data collection forms; data extracted from included studies; data used for all analyses; analytic code; any other materials used in the review. | Supplementary Material |

**Supplementary Table 2.** Literature Search Strategy

| Databases | Key words |
| --- | --- |
| CNKI | 主题：(母乳 OR 人乳) AND 磷脂或摘要检索式：(母乳 OR 人乳) AND 磷脂 |
| Wanfang | 主题:(母乳OR人乳)AND磷脂)or题名或关键词:(母乳OR人乳)AND磷脂) |
| VIP | 题名或关键词:(母乳OR人乳)AND磷脂) |
| Pubmed | ("phospholipid"[Title/Abstract] OR "phosphatidylcholine"[Title/Abstract] OR "phosphatidylethanolamine"[Title/Abstract] OR "phosphatidylinositol"[Title/Abstract] OR "phosphatidylserine"[Title/Abstract] OR "sphingomyelin") AND("breast milk"OR "human milk"OR "mother milk"OR "woman milk"OR "women milk"[Title/Abstract] OR "lactating milk"[Title/Abstract]) |
| Web of Science | All fields:（“phospholipid” OR “phosphatidylcholine” OR “phosphatidylethanolamine” OR “phosphatidylinositol” OR “phosphatidylserine” OR “sphingomyelin”） AND（“breast milk”OR “human milk”OR “mother milk”OR “woman milk”OR “women milk” OR “lactating milk”） |
| Sciencedirect | Title, abstract or keywords:（“phospholipid” OR “phosphatidylcholine” OR “phosphatidylethanolamine” OR “phosphatidylinositol” OR “phosphatidylserine” OR “sphingomyelin”） AND（“breast milk”OR “human milk”OR “mother milk”OR “woman milk”OR “women milk” OR “lactating milk”） |

**Supplementary Table 3.** Quality assessment of included studies

| Study | Type of study | Q1 | Q2 | Q3 | Q4 | Q5 | Q6 | Q7 | Q8 | Total |
| --- | --- | --- | --- | --- | --- | --- | --- | --- | --- | --- |
| Wang et al, 2000 | cross-sectional study | Yes | Yes | No | No | Yes | No | Yes | Yes | 5 |
| Ilcol et al, 2005 | cross-sectional study | Yes | Yes | Yes | Yes | No | No | Yes | Yes | 6 |
| Sala-Vila et al, 2005 | longitudinal study | Yes | Yes | Yes | Yes | Yes | Yes | Yes | Yes | 8 |
| Shoji et al, 2005 | longitudinal study | Yes | Yes | Yes | Yes | Yes | Yes | Yes | Yes | 8 |
| Benoit et al, 2010 | cross-sectional study | Yes | Yes | No | No | No | No | Yes | Yes | 4 |
| Lopez et al, 2011 | cross-sectional study | Yes | Yes | No | No | No | No | Yes | Yes | 4 |
| Garcia et al, 2012 | cross-sectional study | Yes | Yes | Yes | No | Yes | No | Yes | Yes | 6 |
| Giuffrida et al, 2013 | cross-sectional study | Yes | Yes | Yes | No | Yes | No | Yes | Yes | 6 |
| Thakkar et al, 2013 | longitudinal study | Yes | Yes | Yes | Yes | Yes | No | Yes | Yes | 7 |
| Xia, 2015 | longitudinal study | Yes | Yes | Yes | Yes | Yes | No | Yes | Yes | 7 |
| Tavazzi et al, 2017 | cross-sectional study | Yes | Yes | No | No | Yes | No | Yes | Yes | 5 |
| Ma et al, 2017 | cross-sectional study | Yes | Yes | No | Yes | Yes | No | Yes | Yes | 6 |
| McJarrow et al, 2019 | cross-sectional study | Yes | Yes | Yes | Yes | Yes | No | Yes | Yes | 7 |
| Wei et al, 2019 | longitudinal study | Yes | Yes | No | Yes | No | No | Yes | Yes | 5 |
| Wu et al, 2019 | longitudinal study | Yes | Yes | Yes | Yes | Yes | No | Yes | Yes | 7 |
| Fong et al, 2020 | longitudinal study | Yes | Yes | No | Yes | Yes | No | Yes | Yes | 6 |
| Zhao et al, 2020 | longitudinal study | Yes | Yes | Yes | Yes | Yes | Yes | Yes | Yes | 8 |
| Zhou, 2020 | cross-sectional study | Yes | Yes | Yes | Yes | Yes | No | Yes | Yes | 7 |
| Duan et al, 2021 | cross-sectional study | Yes | Yes | No | Yes | Yes | No | Yes | Yes | 6 |
| Liang et al, 2021 | cross-sectional study | Yes | Yes | Yes | Yes | Yes | No | Yes | Yes | 7 |
| Weng, 2021 | cross-sectional study | No | Yes | No | No | Yes | No | Yes | Yes | 4 |
| Lin et al, 2022 | cross-sectional study | No | Yes | Yes | No | No | No | Yes | Yes | 4 |
| Yang et al, 2022 | cross-sectional study | Yes | Yes | Yes | Yes | Yes | Yes | Yes | Yes | 8 |
| Li, 2023 | cross-sectional study | Yes | Yes | Yes | Yes | No | No | Yes | Yes | 6 |
| Zhu et al, 2023 | cross-sectional study | Yes | Yes | Yes | Yes | Yes | No | Yes | Yes | 7 |

Q1.Were the criteria for inclusion in the sample clearly defined?

Q2. Were the study subjects and the setting described in detail？

Q3. Was the sample size adequate?

Q4. Was the exposure measured in a valid and reliable way?

Q5. Were confounding factors identified?

Q6. Were strategies to deal with confounding factors stated?

Q7. Was the outcomes measured in a valid and reliable way ?

Q8. Was appropriate statistical analysis used?

**Supplementary Table 4.** Sensitivity Analysis of Studies on Total Phospholipids in Colostrum

| Study | Mean | Lower 95%CI | Upper 95%CI | p value | I^2^(%) |
| --- | --- | --- | --- | --- | --- |
| (-)Ma et al，2017 | 238.334 | 151.958 | 324.709 | z = 7.661, p = 0.000 | 96.94 |
| (-)Wei et al，2019 | 260.451 | 164.978 | 355.925 | z = 7.574, p = 0.000 | 97.27 |
| (-)Wu et al，2019 | 219.468 | 168.517 | 270.418 | z = 11.959, p = 0.000 | 74.55 |
| (-)Zhao et al，2020 | 261.387 | 166.112 | 356.663 | z = 7.617, p = 0.000 | 97.27 |
| (-)Lin，2022 | 261.391 | 165.106 | 357.676 | z = 7.537, p = 0.000 | 97.27 |
| (-)Yang et al，2022 | 261.580 | 165.106 | 358.053 | z = 7.528, p = 0.000 | 96.01 |
| Pooled effect | 251.596 | 175.616 | 327.577 | z = 8.512, p = 0.000 | 96.59 |

**Supplementary Table 5.** Sensitivity Analysis of Studies on Total Phospholipids in Transitional Milk

| Study | Mean | Lower 95% CI | Upper 95% CI | p value | I^2^(%) |
| --- | --- | --- | --- | --- | --- |
| (-)Ma et al，2017 | 229.536 | 152.173 | 306.899 | z = 7.627, p = 0.000 | 98.79 |
| (-)McJarrow et al，2019 | 229.020 | 151.883 | 306.157 | z = 7.632, p = 0.000 | 98.75 |
| (-)Wei et al，2019 | 241.570 | 165.686 | 317.454 | z = 8.183, p = 0.000 | 98.85 |
| (-)Wu et al，2019 | 211.882 | 167.462 | 256.302 | z = 12.262, p = 0.000 | 91.85 |
| (-)Zhao et al，2020 | 245.144 | 172.735 | 317.553 | z = 8.703, p = 0.000 | 98.85 |
| (-)Lin，2022 | 241.400 | 164.975 | 317.826 | z = 8.120, p = 0.000 | 98.85 |
| (-)Yang et al，2022 | 242.994 | 167.876 | 318.111 | z = 8.315, p = 0.000 | 97.90 |
| Pooled effect | 234.708 | 171.066 | 298.351 | z = 9.024, p = 0.000 | 98.62 |

**Supplementary Table 6.** Sensitivity Analysis of Studies on Total Phospholipids in Mature Milk

| Study | Mean | Lower 95% CI | Upper 95% CI | p value | I^2^(%) |
| --- | --- | --- | --- | --- | --- |
| (-)Garcia et al，2012 | 192.819 | 161.343 | 224.295 | z = 13.347, p = 0.000 | 98.15 |
| (-)Giuffrida et al，2013 | 192.867 | 160.404 | 225.331 | z = 12.944, p = 0.000 | 97.85 |
| (-)Thakkar et al，2013 | 194.535 | 161.203 | 227.866 | z = 12.716, p = 0.000 | 98.11 |
| (-)Ma et al，2017 | 196.887 | 163.122 | 230.652 | z = 12.705, p = 0.000 | 98.19 |
| (-)McJarrow et al，2019 | 195.422 | 162.059 | 228.785 | z = 12.762, p = 0.000 | 98.18 |
| (-)Wei et al，2019 | 198.896 | 165.291 | 232.501 | z = 12.896, p = 0.000 | 98.13 |
| (-)Wu et al，2019 | 189.667 | 160.706 | 218.628 | z = 14.269, p = 0.000 | 97.82 |
| (-)Fong et al，2020 | 195.348 | 161.884 | 228.813 | z = 12.719, p = 0.000 | 98.17 |
| (-)Zhao et al，2020 | 198.447 | 165.324 | 231.571 | z = 13.054, p = 0.000 | 98.18 |
| (-)Duan et al，2021 | 204.659 | 175.925 | 233.394 | z = 15.519, p = 0.000 | 97.11 |
| (-)Liang et al，2021 | 198.834 | 165.036 | 232.631 | z = 12.818, p = 0.000 | 97.98 |
| (-)Weng，2021 | 203.649 | 174.918 | 232.380 | z = 15.444, p = 0.000 | 98.07 |
| (-)Lin，2022 | 198.877 | 165.285 | 232.470 | z = 12.899, p = 0.000 | 98.14 |
| (-)Yang et al，2022 | 197.170 | 162.972 | 231.368 | z = 12.562, p = 0.000 | 98.08 |
| Pooled effect | 197.003 | 166.075 | 227.930 | z = 13.761, p = 0.000 | 98.04 |

**Supplementary Table 7.** Sensitivity Analysis of Studies on SM in Colostrum

| Study | Mean | Lower 95% CI | Upper 95% CI | p value | I^2^(%) |
| --- | --- | --- | --- | --- | --- |
| (-)Ilcol et al，2005 | 63.782 | 41.648 | 85.917 | z = 7.051, p = 0.000 | 97.72 |
| (-)Ma et al，2017 | 72.550 | 50.602 | 94.499 | z = 8.088, p = 0.000 | 99.27 |
| (-)Wei et al，2019 | 68.809 | 43.814 | 93.804 | z = 6.736, p = 0.000 | 99.27 |
| (-)Wu et al，2019 | 63.000 | 42.702 | 83.299 | z = 7.595, p = 0.000 | 99.06 |
| (-)Zhao et al，2020 | 66.364 | 42.055 | 90.672 | z = 6.680, p = 0.000 | 99.26 |
| (-)Lin，2022 | 68.068 | 42.998 | 93.138 | z = 6.644, p = 0.000 | 99.24 |
| (-)Yang et al，2022 | 71.506 | 48.070 | 94.943 | z = 7.466, p = 0.000 | 98.18 |
| (-)Li，2023 | 71.842 | 48.838 | 94.847 | z = 7.642, p = 0.000 | 99.28 |
| Pooled effect | 68.262 | 47.283 | 89.241 | z = 7.694, p = 0.000 | 99.16 |

**Supplementary Table 8.** Sensitivity Analysis of Studies on SM in Transitional Milk

| Study | Mean | Lower 95% CI | Upper 95% CI | p value | I^2^(%) |
| --- | --- | --- | --- | --- | --- |
| (-)Ma et al，2017 | 69.853 | 47.800 | 91.906 | z = 7.750, p = 0.000 | 99.18 |
| (-)McJarrow et al，2019 | 60.004 | 34.697 | 85.311 | z = 5.802, p = 0.000 | 99.20 |
| (-)Wei et al，2019 | 64.003 | 36.457 | 91.549 | z = 5.685, p = 0.000 | 99.31 |
| (-)Wu et al，2019 | 57.370 | 35.993 | 78.748 | z = 6.567, p = 0.000 | 98.41 |
| (-)Zhao et al，2020 | 62.213 | 35.123 | 89.302 | z = 5.619, p = 0.000 | 99.29 |
| (-)Lin，2022 | 63.572 | 36.005 | 91.139 | z = 5.643, p = 0.000 | 99.29 |
| (-)Yang et al，2022 | 67.017 | 40.812 | 93.223 | z = 6.258, p = 0.000 | 99.27 |
| (-)Li，2023 | 66.996 | 40.805 | 93.188 | z = 6.259, p = 0.000 | 99.32 |
| Pooled effect | 63.890 | 40.802 | 86.977 | z = 6.544, p = 0.000 | 99.21 |

**Supplementary Table 9.** Sensitivity Analysis of Studies on SM in Mature Milk

| Study | Mean | Lower 95% CI | Upper 95% CI | p value | I^2^(%) |
| --- | --- | --- | --- | --- | --- |
| (-)Ilcol et al，2005 | 70.564 | 60.018 | 81.110 | z = 14.185, p = 0.000 | 99.20 |
| (-)Garcia et al，2012 | 69.990 | 59.560 | 80.420 | z = 14.226, p = 0.000 | 99.32 |
| (-)Giuffrida et al，2013 | 69.488 | 59.214 | 79.761 | z = 14.339, p = 0.000 | 99.27 |
| (-)Thakkar et al，2013 | 69.782 | 59.378 | 80.186 | z = 14.219, p = 0.000 | 99.27 |
| (-)Ma et al，2017 | 70.340 | 59.823 | 80.857 | z = 14.178, p = 0.000 | 99.32 |
| (-)Tavazzi et al，2017 | 69.531 | 59.396 | 79.666 | z = 14.544, p = 0.000 | 99.32 |
| (-)McJarrow et al，2019 | 69.844 | 59.455 | 80.233 | z = 14.252, p = 0.000 | 99.31 |
| (-)Wei et al，2019 | 71.017 | 60.528 | 81.506 | z = 14.353, p = 0.000 | 99.32 |
| (-)Wu et al，2019 | 69.143 | 59.080 | 79.205 | z = 14.567, p = 0.000 | 99.27 |
| (-)Fong et al，2020 | 70.616 | 60.084 | 81.147 | z = 14.214, p = 0.000 | 99.32 |
| (-)Zhao et al，2020 | 70.703 | 60.215 | 81.190 | z = 14.292, p = 0.000 | 99.32 |
| (-)Zhou，2020 | 67.875 | 59.040 | 76.711 | z = 16.285, p = 0.000 | 98.85 |
| (-)Liang et al，2021 | 72.070 | 62.049 | 82.091 | z = 15.246, p = 0.000 | 99.25 |
| (-)Duan et al，2021 | 71.632 | 61.374 | 81.890 | z = 14.803, p = 0.000 | 99.33 |
| (-)Weng，2021 | 72.211 | 62.594 | 81.828 | z = 15.918, p = 0.000 | 99.32 |
| (-)Lin，2022 | 70.925 | 60.430 | 81.421 | z = 14.325, p = 0.000 | 99.32 |
| (-)Yang et al，2022 | 71.600 | 61.283 | 81.916 | z = 14.713, p = 0.000 | 99.25 |
| (-)Li，2023 | 72.298 | 62.494 | 82.101 | z = 15.634, p = 0.000 | 99.32 |
| Pooled effect | 70.544 | 60.670 | 80.418 | z = 15.073, p = 0.000 | 99.28 |

**Supplementary Table 10.** Sensitivity Analysis of Studies on PE in Colostrum

| Study | Mean | Lower 95% CI | Upper 95% CI | p value | I^2^(%) |
| --- | --- | --- | --- | --- | --- |
| (-)Ma et al，2017 | 55.277 | 49.974 | 60.580 | z = 26.795, p = 0.000 | 90.77 |
| (-)Wei et al，2019 | 59.806 | 46.253 | 73.359 | z = 11.343, p = 0.000 | 91.69 |
| (-)Wu et al，2019 | 60.804 | 49.012 | 72.597 | z = 13.255, p = 0.000 | 89.53 |
| (-)Zhao et al，2020 | 59.528 | 46.761 | 72.296 | z = 11.985, p = 0.000 | 93.38 |
| (-)Lin et al，2022 | 59.242 | 45.765 | 72.720 | z = 11.299, p = 0.000 | 93.34 |
| (-)Yang et al，2022 | 58.435 | 44.635 | 72.234 | z = 10.885, p = 0.000 | 89.45 |
| (-)Li，2023 | 59.267 | 45.897 | 72.638 | z = 11.395, p = 0.000 | 93.37 |
| Pooled effect | 58.832 | 48.068 | 69.596 | z = 13.374, p = 0.000 | 92.27 |

**Supplementary Table 11.** Sensitivity Analysis of Studies on PE in Transitional Milk

| Study | Mean | Lower 95% CI | Upper 95% CI | p value | I^2^(%) |
| --- | --- | --- | --- | --- | --- |
| (-)Ma et al，2017 | 55.718 | 48.941 | 62.496 | z = 20.115, p = 0.000 | 92.08 |
| (-)McJarrow et al，2019 | 56.070 | 47.124 | 65.016 | z = 15.336, p = 0.000 | 92.49 |
| (-)Wei et al，2019 | 58.143 | 48.717 | 67.569 | z = 15.094, p = 0.000 | 89.11 |
| (-)Wu et al，2019 | 58.507 | 49.840 | 67.173 | z = 16.519, p = 0.000 | 87.83 |
| (-)Zhao et al，2020 | 58.408 | 50.440 | 66.376 | z = 17.937, p = 0.000 | 91.83 |
| (-)Lin et al，2022 | 57.717 | 47.976 | 67.458 | z = 14.498, p = 0.000 | 91.85 |
| (-)Yang et al，2022 | 56.313 | 46.878 | 65.747 | z = 14.605, p = 0.000 | 81.15 |
| (-)Li，2023 | 56.956 | 47.109 | 66.802 | z = 14.154, p = 0.000 | 92.56 |
| Pooled effect | 57.228 | 49.217 | 65.239 | z = 16.892, p = 0.000 | 91.38 |

**Supplementary Table 12.** Sensitivity Analysis of Studies on PE in Mature Milk

| Study | Mean | Lower 95% CI | Upper 95% CI | p value | I2(%) |
| --- | --- | --- | --- | --- | --- |
| (-)Garcia et al，2012 | 57.604 | 47.523 | 67.686 | z = 12.179, p = 0.000 | 99.97 |
| (-)Giuffrida et al，2013 | 56.398 | 46.437 | 66.358 | z = 12.069, p = 0.000 | 99.97 |
| (-)Thakkar et al，2013 | 56.353 | 46.406 | 66.299 | z = 12.076, p = 0.000 | 99.97 |
| (-)Ma et al，2017 | 56.660 | 46.607 | 66.713 | z = 12.013, p = 0.000 | 99.97 |
| (-)Tavazzi et al，2017 | 56.176 | 46.363 | 65.990 | z = 12.201, p = 0.000 | 99.97 |
| (-)McJarrow et al，2019 | 55.820 | 46.216 | 65.425 | z = 12.388, p = 0.000 | 99.97 |
| (-)Wei et al，2019 | 57.574 | 47.474 | 67.673 | z = 12.150, p = 0.000 | 99.97 |
| (-)Wu et al，2019 | 58.465 | 48.735 | 68.195 | z = 12.807, p = 0.000 | 99.97 |
| (-)Fong et al，2020 | 55.701 | 46.168 | 65.235 | z = 12.454, p = 0.000 | 99.97 |
| (-)Zhao et al，2020 | 57.183 | 47.074 | 67.292 | z = 12.057, p = 0.000 | 99.97 |
| (-)Zhou，2020 | 55.724 | 46.162 | 65.286 | z = 12.421, p = 0.000 | 99.97 |
| (-)Duan et al，2021 | 59.142 | 50.630 | 67.655 | z = 14.808, p = 0.000 | 99.01 |
| (-)Liang et al，2021 | 57.399 | 47.275 | 67.524 | z = 12.084, p = 0.000 | 99.97 |
| (-)Weng et al，2021 | 58.992 | 49.724 | 68.261 | z = 13.566, p = 0.000 | 99.97 |
| (-)Lin et al，2022 | 57.740 | 47.681 | 67.800 | z = 12.234, p = 0.000 | 99.97 |
| (-)Yang et al，2022 | 57.000 | 46.888 | 67.113 | z = 12.014, p = 0.000 | 99.94 |
| (-)Li，2023 | 58.275 | 48.436 | 68.114 | z = 12.624, p = 0.000 | 99.97 |
| Pooled effect | 57.208 | 47.746 | 66.669 | z = 12.818, p = 0.000 | 99.97 |

**Supplementary Table 13.** Sensitivity Analysis of Studies on PC in Colostrum

| Study | Mean | Lower 95% CI | Upper 95% CI | p value | I^2^(%) |
| --- | --- | --- | --- | --- | --- |
| (-)Ilcol et al，2005 | 55.153 | 48.408 | 61.898 | z = 21.019, p = 0.000 | 66.39 |
| (-)Ma et al，2017 | 64.112 | 39.396 | 88.829 | z = 6.668, p = 0.000 | 98.74 |
| (-)Wei et al，2019 | 65.974 | 41.228 | 90.720 | z = 6.853, p = 0.000 | 98.75 |
| (-)Zhao et al，2020 | 67.617 | 44.155 | 91.078 | z = 7.408, p = 0.000 | 98.74 |
| (-)Lin et al，2022 | 64.919 | 39.881 | 89.958 | z = 6.665, p = 0.000 | 98.72 |
| (-)Yang et al，2022 | 66.883 | 42.701 | 91.064 | z = 7.110, p = 0.000 | 97.96 |
| (-)Li，2023 | 66.739 | 42.446 | 91.032 | z = 7.062, p = 0.000 | 98.75 |
| Pooled effect | 64.722 | 44.560 | 84.883 | z = 7.855, p = 0.000 | 98.50 |

**Supplementary Table 14.** Sensitivity Analysis of Studies on PC in Transitional Milk

| Study | Mean | Lower 95% CI | Upper 95% CI | p value | I^2^(%) |
| --- | --- | --- | --- | --- | --- |
| (-)Ma et al，2017 | 50.763 | 41.188 | 60.339 | z = 13.628, p = 0.000 | 89.74 |
| (-)McJarrow et al，2019 | 48.627 | 42.029 | 55.224 | z = 18.946, p = 0.000 | 84.85 |
| (-)Wei et al，2019 | 49.970 | 40.647 | 59.293 | z = 13.779, p = 0.000 | 88.54 |
| (-)Zhao et al，2020 | 52.365 | 44.216 | 60.514 | z = 16.518, p = 0.000 | 89.77 |
| (-)Lin et al，2022 | 49.132 | 40.373 | 57.890 | z = 14.420, p = 0.000 | 82.52 |
| (-)Yang et al，2022 | 51.985 | 42.726 | 61.245 | z = 14.432, p = 0.000 | 83.23 |
| (-)Li，2023 | 51.628 | 42.198 | 61.059 | z = 14.073, p = 0.000 | 90.08 |
| Pooled effect | 50.601 | 42.886 | 58.315 | z = 16.050, p = 0.000 | 88.15 |

**Supplementary Table 15.** Sensitivity Analysis of Studies on PC in Mature Milk

| Study | Mean | Lower 95% CI | Upper 95% CI | p value | I^2^(%) |
| --- | --- | --- | --- | --- | --- |
| (-)Ilcol et al，2005 | 47.361 | 31.973 | 62.749 | z = 6.560, p = 0.000 | 98.64 |
| (-)Garcia et al，2012 | 49.275 | 32.854 | 65.695 | z = 6.396, p = 0.000 | 99.26 |
| (-)Giuffrida et al，2013 | 49.303 | 32.798 | 65.808 | z = 6.367, p = 0.000 | 99.23 |
| (-)Thakkar et al，2013 | 49.962 | 33.324 | 66.599 | z = 6.401, p = 0.000 | 99.25 |
| (-)Ma et al，2017 | 51.574 | 35.263 | 67.886 | z = 6.739, p = 0.000 | 99.24 |
| (-)Tavazzi et al，2017 | 50.053 | 33.516 | 66.590 | z = 6.451, p = 0.000 | 99.27 |
| (-)McJarrow et al，2019 | 51.306 | 34.955 | 67.656 | z = 6.688, p = 0.000 | 99.27 |
| (-)Wei et al，2019 | 50.373 | 33.709 | 67.038 | z = 6.443, p = 0.000 | 99.26 |
| (-)Wu et al，2019 | 44.313 | 35.797 | 52.829 | z = 11.091, p = 0.000 | 99.09 |
| (-)Fong et al，2020 | 50.268 | 33.660 | 66.876 | z = 6.451, p = 0.000 | 99.27 |
| (-)Zhao et al，2020 | 51.159 | 34.762 | 67.557 | z = 6.650, p = 0.000 | 99.27 |
| (-)Duan et al，2021 | 51.835 | 35.701 | 67.970 | z = 6.848, p = 0.000 | 99.13 |
| (-)Liang et al，2021 | 51.138 | 34.255 | 68.021 | z = 6.456, p = 0.000 | 99.27 |
| (-)Weng et al，2021 | 51.654 | 35.478 | 67.830 | z = 6.806, p = 0.000 | 99.26 |
| (-)Lin et al，2022 | 50.266 | 33.634 | 66.898 | z = 6.442, p = 0.000 | 99.27 |
| (-)Yang et al，2022 | 51.263 | 34.198 | 68.329 | z = 6.403, p = 0.000 | 99.26 |
| (-)Li，2023 | 50.935 | 34.409 | 67.461 | z = 6.569, p = 0.000 | 99.27 |
| Pooled effect | 50.089 | 34.587 | 65.591 | z = 6.850, p = 0.000 | 99.22 |

**Supplementary Table 16.** Sensitivity Analysis of Studies on PS in Colostrum

| Study | Mean | Lower 95% CI | Upper 95% CI | p value | I^2^(%) |
| --- | --- | --- | --- | --- | --- |
| (-)Wei et al，2019 | 14.842 | -10.655 | 40.339 | z = 2.505, p = 0.012 | 99.95 |
| (-)Lin et al，2022 | 14.856 | -10.607 | 40.318 | z = 2.510, p = 0.012 | 99.97 |
| (-)Yang et al，2022 | 9.319 | 5.949 | 12.690 | z = 11.896, p = 0.000 | 93.94 |
| (-)Li，2023 | 15.627 | -8.024 | 39.277 | z = 2.843, p = 0.004 | 99.95 |
| Pooled effect | 13.669 | -0.179 | 27.516 | z = 3.141, p = 0.002 | 99.96 |

**Supplementary Table 17.** Sensitivity Analysis of Studies on PS in Transitional Milk

| Study | Mean | Lower 95% CI | Upper 95% CI | p value | I^2^(%) |
| --- | --- | --- | --- | --- | --- |
| (-)McJarrow et al，2019 | 10.966 | 3.887 | 18.045 | z = 4.930, p = 0.000 | 99.62 |
| (-)Wei et al，2019 | 15.408 | 0.439 | 30.376 | z = 3.276, p = 0.001 | 99.58 |
| (-)Lin et al，2022 | 15.520 | 0.640 | 30.400 | z = 3.319, p = 0.001 | 99.41 |
| (-)Yang et al，2022 | 12.859 | -1.903 | 27.622 | z = 2.772, p = 0.006 | 96.89 |
| (-)Li，2023 | 15.863 | 1.744 | 29.981 | z = 3.576, p = 0.000 | 99.34 |
| Pooled effect | 14.209 | 3.596 | 24.822 | z = 3.717, p = 0.000 | 99.51 |

**Supplementary Table 18.** Sensitivity Analysis of Studies on PS in Mature Milk

| Study | Mean | Lower 95% CI | Upper 95% CI | p value | I^2^(%) |
| --- | --- | --- | --- | --- | --- |
| (-)Garcia et al，2012 | 11.757 | 7.963 | 15.551 | z = 6.905, p = 0.000 | 99.93 |
| (-)Giuffrida et al，2013 | 12.530 | 8.076 | 16.985 | z = 6.268, p = 0.000 | 99.81 |
| (-)Thakkar et al，2013 | 13.023 | 8.715 | 17.331 | z = 6.736, p = 0.000 | 99.93 |
| (-)Ma et al，2017 | 12.136 | 7.857 | 16.415 | z = 6.319, p = 0.000 | 99.93 |
| (-)McJarrow et al，2019 | 12.293 | 7.936 | 16.650 | z = 6.287, p = 0.000 | 99.93 |
| (-)Wei et al，2019 | 13.030 | 8.724 | 17.337 | z = 6.742, p = 0.000 | 99.93 |
| (-)Fong et al，2020 | 12.899 | 8.530 | 17.269 | z = 6.577, p = 0.000 | 99.93 |
| (-)Liang et al，2021 | 12.190 | 7.869 | 16.511 | z = 6.286, p = 0.000 | 99.93 |
| (-)Weng et al，2021 | 13.567 | 9.820 | 17.314 | z = 8.068, p = 0.000 | 99.93 |
| (-)Lin et al，2022 | 13.042 | 8.744 | 17.341 | z = 6.760, p = 0.000 | 99.93 |
| (-)Yang et al，2022 | 11.857 | 7.791 | 15.924 | z = 6.497, p = 0.000 | 99.36 |
| (-)Li，2023 | 12.970 | 8.631 | 17.310 | z = 6.659, p = 0.000 | 99.93 |
| Pooled effect | 12.601 | 8.608 | 16.595 | z = 6.945, p = 0.000 | 99.93 |

**Supplementary Table 19.** Sensitivity Analysis of Studies on PI in Colostrum

| Study | Mean | Lower 95% CI | Upper 95% CI | p value | I^2^(%) |
| --- | --- | --- | --- | --- | --- |
| (-)Ma et al，2017 | 9.645 | 0.650 | 18.639 | z = 2.977, p = 0.003 | 99.93 |
| (-)Wei et al，2019 | 10.859 | 1.349 | 20.368 | z = 3.170, p = 0.002 | 99.81 |
| (-)Zhao et al，2020 | 11.510 | 2.516 | 20.504 | z = 3.553, p = 0.000 | 99.93 |
| (-)Lin et al，2022 | 11.027 | 1.594 | 20.460 | z = 3.245, p = 0.001 | 99.92 |
| (-)Yang et al，2022 | 7.915 | 2.648 | 13.181 | z = 4.173, p = 0.000 | 97.07 |
| (-)Li，2023 | 12.036 | 3.889 | 20.184 | z = 4.102, p = 0.000 | 99.90 |
| Pooled effect | 10.553 | 3.323 | 17.782 | z = 3.752, p = 0.000 | 99.91 |

**Supplementary Table 20.** Sensitivity Analysis of Studies on PI in Transitional Milk

| Study | Mean | Lower 95% CI | Upper 95% CI | p value | I^2^(%) |
| --- | --- | --- | --- | --- | --- |
| (-)Ma et al，2017 | 10.144 | 3.280 | 17.009 | z = 4.103, p = 0.000 | 99.65 |
| (-)McJarrow et al，2019 | 10.144 | 3.278 | 17.009 | z = 4.102, p = 0.000 | 99.65 |
| (-)Wei et al，2019 | 10.772 | 4.057 | 17.486 | z = 4.454, p = 0.000 | 99.64 |
| (-)Zhao et al，2020 | 11.432 | 5.647 | 17.218 | z = 5.486, p = 0.000 | 99.42 |
| (-)Lin et al，2022 | 10.887 | 4.263 | 17.511 | z = 4.563, p = 0.000 | 99.49 |
| (-)Yang et al，2022 | 8.494 | 5.105 | 11.883 | z = 6.959, p = 0.000 | 92.28 |
| Pooled effect | 10.320 | 5.108 | 15.533 | z = 5.090, p = 0.000 | 99.59 |

**Supplementary Table 21.** Sensitivity Analysis of Studies on PI in Mature Milk

| Study | Mean | Lower 95% CI | Upper 95% CI | p value | I^2^(%) |
| --- | --- | --- | --- | --- | --- |
| (-)Garcia et al，2012 | 10.137 | 7.171 | 13.103 | z = 7.522, p = 0.000 | 99.82 |
| (-)Giuffrida et al，2013 | 10.154 | 7.183 | 13.126 | z = 7.521, p = 0.000 | 99.82 |
| (-)Thakkar et al，2013 | 10.024 | 7.093 | 12.956 | z = 7.525, p = 0.000 | 99.82 |
| (-)Ma et al，2017 | 10.521 | 7.612 | 13.430 | z = 7.960, p = 0.000 | 99.79 |
| (-)Tavazzi et al，2017 | 10.212 | 7.241 | 13.182 | z = 7.567, p = 0.000 | 99.83 |
| (-)McJarrow et al，2019 | 10.560 | 7.676 | 13.445 | z = 8.058, p = 0.000 | 99.81 |
| (-)Wei et al，2019 | 10.526 | 7.621 | 13.432 | z = 7.974, p = 0.000 | 99.80 |
| (-)Fong et al，2020 | 10.477 | 7.548 | 13.407 | z = 7.872, p = 0.000 | 99.81 |
| (-)Zhao et al，2020 | 10.696 | 7.915 | 13.477 | z = 8.466, p = 0.000 | 99.81 |
| (-)Zhou，2020 | 10.235 | 7.255 | 13.215 | z = 7.560, p = 0.000 | 99.78 |
| (-)Liang et al，2021 | 9.586 | 7.059 | 12.113 | z = 8.348, p = 0.000 | 99.83 |
| (-)Lin et al，2022 | 10.586 | 7.718 | 13.455 | z = 8.123, p = 0.000 | 99.80 |
| (-)Yang et al，2022 | 9.490 | 7.098 | 11.882 | z = 8.733, p = 0.000 | 99.21 |
| Pooled effect | 10.247 | 7.534 | 12.960 | z = 8.230, p = 0.000 | 99.81 |

**Supplementary Table 22.** Subgroup Analysis of SM in Mature Milk

|  | Subgroup | Mean（95%CI） | Heterogeneity(I^2^) | P Value |
| --- | --- | --- | --- | --- |
| Economic level | Developed | 78.77(59.30，98.24) | 96.69 | 0.22 |
|  | Developing | 67.59(54.95，80.23) | 99.38 |  |
| Geographical location | China | 65.21 (48.47，81.95) | 99.40 | 0.17 |
|  | Non-China | 77.15(66.79，87.50) | 95.11 |  |
| Detection methods | 31P NMR | 64.66(45.50，83.81) | 96.16 | 0.13 |
|  | HPLC-ELSD | 60.24(12.41，108.07) | 99.48 |  |
|  | LC-MS | 83.08 (65.12，101.05) | 97.85 |  |
|  | Other | 68.57(4.54，132.60) | 99.04 |  |

**Supplementary Table 23.** Subgroup Analysis of PE in Mature Milk

|  | Subgroup | Mean（95%CI） | Heterogeneity(I^2^) | P Value |
| --- | --- | --- | --- | --- |
| Economic level | Developed | 53.06 (15.53，90.59) | 99.99 | 0.70 |
|  | Developing | 57.94(47.52，68.35) | 97.65 |  |
| Geographical location | China | 54.18 (47.88，60.48) | 98.18 | 0.58 |
|  | Non-China | 61.74(35.72，87.76) | 99.98 |  |
| Detection methods | 31P NMR | 45.03(26.27，63.84) | 99.98 | 0.00 |
|  | HPLC-ELSD | 61.60(41.57，81.63) | 97.52 |  |
|  | LC-MS | 74.25(64.57，83.93) | 76.39 |  |
|  | Other | 38.22(22.244，54.20) | 0.00 |  |

**Supplementary Table 24.** Subgroup Analysis of PC in Mature Milk

|  | Subgroup | Mean（95%CI） | Heterogeneity(I^2^) | P Value |
| --- | --- | --- | --- | --- |
| Economic level | Developed | 50.48(30.00，70.95) | 99.21 | 0.97 |
|  | Developing | 50.06(27.94，72.18) | 99.28 |  |
| Geographical location | China | 49.34(22.69，75.98) | 98.34 | 0.98 |
|  | Non-China | 49.07(31.78，66.37) | 99.55 |  |
| Detection methods | 31P NMR | 44.61(25.98，63.24) | 98.98 | 0.33 |
|  | HPLC-ELSD | 44.03 (27.62，60.43) | 96.67 |  |
|  | LC-MS | 37.34 (24.26，50.43) | 92.87 |  |
|  | Other | 92.31(-60.28，244.89) | 99.63 |  |

**Supplementary Table 25.** Subgroup Analysis of PS in Mature Milk

|  | Subgroup | Mean（95%CI） | Heterogeneity(I^2^) | P Value |
| --- | --- | --- | --- | --- |
| Economic level | Developed | 14.88(-4.16，33.91) | 99.65 | 0.54 |
|  | Developing | 11.88(7.189，16.57) | 99.75 |  |
| Geographical location | China | 10.45(4.72，16.19) | 99.81 | 0.13 |
|  | Non-China | 15.68(8.80，22.56) | 99.33 |  |
| Detection methods | 31P NMR | 10.89 (0.91，20.87) | 99.47 | 0.05 |
|  | HPLC-ELSD | 15.13 (-0.75，31.01) | 99.91 |  |
|  | LC-MS | 14.37(3.25，25.48) | 97.07 |  |
|  | Other | 8.60(7.67，9.53) | - |  |

**Supplementary Table 26.** Subgroup Analysis of PI in Mature Milk

|  | Subgroup | Mean（95%CI） | Heterogeneity(I^2^) | P Value |
| --- | --- | --- | --- | --- |
| Economic level | Developed | 11.91(10.49，13.33) | 51.79 | 0.21 |
|  | Developing | 9.64(5.55，13.73) | 99.86 |  |
| Geographical location | China | 10.46(5.06，15.87) | 99.85 | 0.84 |
|  | Non-China | 9.95 (7.13，12.77) | 96.63 |  |
| Detection methods | 31P NMR | 8.95 (4.43，13.48) | 96.60 | 0.00 |
|  | HPLC-ELSD | 16.78 (8.51，25.06) | 98.89 |  |
|  | LC-MS | 7.66(5.35，9.98) | 96.35 |  |
